
JOURNAL INFORMATION
==============================
NLM Title Abbreviation: ScientificWorldJournal
Journal ID: ScientificWorldJournal
NLM Journal ID: 101131163
Journal ID: TSWJ

The Scientific World Journal

ISSN: 2356-6140
EISSN: 1537-744X
Publisher: Wiley

ARTICLE INFORMATION
==============================
PMCID: PMC5900483
PMID: 17370024
DOI: 10.1100/tsw.2007.91
Article ID: 413019
Article version: 1
Subjects: Research Article

Global Warming 2007. An Update to Global Warming: The Balance of Evidence and Its Policy Implications

Keller Charles F. *
Institute of Geophysics and Planetary Physics, Los Alamos National Laboratory, Los Alamos, NM, USA
*Corresponding author.Academic Editor: Howard P. Hanson

Publication date: 2007
Electronic publication date: 2007 Mar 9
Volume: 7
First page: 381
Last page: 399
Received 2007 Feb 22; Accepted 2007 Feb 25
Copyright: Copyright © 2007 Charles F. Keller.
Copyright year: 2007
License: This is an open access article distributed under the Creative Commons Attribution License, which permits unrestricted use, distribution, and reproduction in any medium, provided the original work is properly cited.
License URL: https://creativecommons.org/licenses/by/3.0/

Keywords: climate, climate change, global warming, climate modeling, atmosphere, ocean, greenhouse gases, carbon dioxide, solar activity, environment, ecosystems

==============================
Research Article
*TheScientificWorldJOURNAL,* (2007) **7**, 381–399
ISSN 1537-744X; DOI 10.1100/tsw.2007.91

TheScientificWorldJOURNAL
www.thescientificworld.com

### /Global Warming 2007
### *An Update to Global Warming: The Balance of Evidence and Its Policy Implications*

Charles F. Keller

*Institute of Geophysics and Planetary Physics, Los Alamos National Laboratory, Los Alamos, NM, USA*

**E-mail:** alfanso@mail.cybermesa.com

*Received February 22, 2007; Accepted February 25, 2007; Published March 9, 2007*

**This is an update to the research article published, Vol. 3**

**In the four years since my original review (Keller[25]; hereafter referred to as CFK03), research has clarified and strengthened our understanding of how humans are warming the planet. So many of the details highlighted in the IPCC's Third Assessment Report[21] and in CFK03 have been resolved that I expect many to be a bit overwhelmed, and I hope that, by treating just the most significant aspects of the research, this update may provide a road map through the expected maze of new information. In particular, while most of CFK03 remains current, there are important items that have changed:**

- **Most notable is the resolution of the conundrum that mid-tropospheric warming did not seem to match surface warming. Both satellite and radiosonde (balloon-borne sensors) data reduction showed little warming in the middle troposphere (4-8 km altitude). In the CFK03 I discussed potential solutions to this problem, but at that time there was no clear resolution. This problem has now been solved, and the middle troposphere is seen to be warming apace with the surface.**
- **There have also been advances in determinations of temperatures over the past 1,000 years showing a cooler Little Ice Age (LIA) but essentially the same warming during medieval times (not as large as recent warming). The recent uproar over the so-called "hockey stick" temperature determination is much overblown since at least seven other groups have made relatively independent determinations of northern hemisphere temperatures over the same time period and derived essentially the same results. They differ on how cold the LIA was but essentially agree with the Mann's hockey stick result that the Medieval Warm Period was not as warm as the last 25 years.**
- **The question of the sun's influence on climate continues to generate controversy. It appears there is a growing consensus that, while the sun was a major factor in earlier temperature variations, it is incapable of having caused**

©2007 with author.
Published by TheScientificWorld; www.thescientificworld.com

**observed warming in the past quarter century or so. However, this conclusion is being challenged by differing interpretations of satellite observations of Total Solar Insolation (TSI). Different satellites give different estimates of TSI during the 1996-7 solar activity minimum. A recent study using the larger TSI satellite interpretation indicates a stronger role for the sun, and until there is agreement on TSI at solar minimum, we caution completely disregarding the sun as a significant factor in recent warming.**

- **Computer models continue to improve and, while they still do not do a satisfactory job of predicting regional changes, their simulations of global aspects of climate change and of individual forcings are increasingly reliable.**
- **In addition to these four areas, the past five years have seen advances in our understanding of many other aspects of climate change—from albedo changes due to land use to the dynamics of glacier movement. However, these more are of second order importance and will only be treated very briefly.**

**The big news since CFK03 is the first of these, the collapse of the climate critics' last real bastion, namely that satellites and radiosondes show no significant warming in the past quarter century. Figuratively speaking, this was the center pole that held up the critics' entire "tent." Their argument was that, if there had been little warming in the past 25 years or so, then what warming was observed would have been within the range of natural variations with solar forcing as the major player. Further, the models would have been shown to be unreliable since they were predicting warming that was not happening. But now both satellite and *in-situ* radiosonde observations have been shown to corroborate both the surface observations of warming and the model predictions.**

**Thus, while uncertainties still remain, we are now seeing a coherent picture in which past climate variations, solar and other forcings, model predictions and other indicators such as glacier recession all point to a human-induced warming that needs to be considered carefully.**

**A final topic touched on briefly here is the new understanding of the phenomenon called "global dimming." Several sets of observations of the sun's total radiation at the surface have shown that there has been a reduction in sunlight reaching it. This has been related to the scattering of sunlight by aerosols and has led to a better quantification of the possibility that cleaning up our atmospheric pollution will lead to greater global warming.**

**Adding all these advances together, there is a growing consensus that the 21st century will indeed see some 2°C (3.5°F) or more in additional warming. This is corroborated in the new IPCC Assessment, an early release of which is touched on very briefly here.**

**KEY WORDS:** climate, climate change, global warming, climate modeling, atmosphere, ocean, greenhouse gases, carbon dioxide, solar activity, environment, ecosystems

### INTRODUCTION

Since CFK03, there have been significant advances in our understanding of climate change and the degree to which humans are affecting it. This update will attempt to describe succinctly the most salient of these advances partly as a simplifying preparation for more easily understanding the upcoming Fourth Assessment Report (4AR) of the IPCC, the full version of which is to come out sometime in mid-2007.

The subject of climate change and human influences on it is extremely complicated and has a seeming endless number of areas that are being studied. In addition, the small but well-focused objections to the mainstream consensus (that human influence is becoming a dominant climate driver) provide further confusion. Most of these objections and the additional research they have stimulated are what I would call “second order considerations”; that is, they are issues that are of secondary importance to whether or not human emissions of so-called greenhouse gases (GHGs) are causing substantial warming.

Many of these issues, such as changes in land use that affect the earth's reflectivity, are indeed important for us to understand in order to more accurately decide what to do about global warming. But they are not what you would call "show stoppers." Too often critics of anthropogenic warming in general or proponents of these secondary issues would have people believe that, since we do not understand these issues well enough, we cannot be certain of the overall human influence. It is this propensity that caused me in a perhaps incautious interview statement to call these people excellent scientists but nonetheless "nit pickers." They represent a class of people every scientist knows about, people who can always point out what is wrong with an idea or a hypothesis. To the extent that this causes the rest of us to sharpen our thinking and focus our research, it has real value. But, when the nay-saying leads to widespread thinking that uncertainties are large enough to invalidate a well-established idea, the nit-picking approach needs to be seen for what it is and, while noted, not as anything major.

To me there are only a few "first order" questions. These are the ones I dealt with at length in CFK03. To repeat, they are the following:

1) Is the observed temperature increase since the 1960s real? If it is,
2) Is it beyond what might be expected by natural variability? If it is,
3) Can we understand it and predict its future magnitude using both data reduction and large-scale computer models?
4) If we can, we have established a major influence on climate by humans and the remaining myriad of related issues, while important for increasing our understanding, are of second order and will not change the answer to the basic question of whether humans are substantially warming the earth by emitting GHGs.

This update will be divided into five sections, the above four with the second divided into two parts—recent paleoclimate findings and solar influences. In this paper I will assume that the reader is already familiar with CFK03, which contains the essential background and state of the art in early 2003 and will be necessary for a fuller understanding of this update.

### IS THE SURFACE TEMPERATURE RECORD ACCURATE?

In CFK03, I discussed the problem that, while the surface record showed temperatures since the late 1970s rising rapidly to levels probably not seen in thousands of years, both satellite and balloon-borne (radiosonde) temperature data seemed to show little or no warming in the middle troposphere. If this were so, then several alternatives were possible:

1) The surface record was wrong, infected by a wide variety of sampling inaccuracies from urban heat island effects to incorrect determinations of ocean surface temperatures.
2) Some unknown atmospheric effects were altering the vertical lapse rate of temperature with altitude such that the middle troposphere was not participating in the surface warming.

3) The large observed cooling of the lower stratosphere was cooling the upper troposphere masking the warming lower down.
4) There was something wrong with both satellite and balloon data reductions.

In CFK03, I dealt with the first three of these and touched on the fourth, which presented a real dilemma because there appeared to be two independent records both showing little warming. One problem was that the satellite record had been studied by only a single team, that of John Christy and Roy Spencer[8,9] at the NASA / University of Alabama site in Huntsville (referred to hereafter as UAH). Since that time several other groups began re-examining this data and discovering various matters to be concerned about, from changes in the upper atmosphere with changing solar cycle to satellite orbit drifts. One of these groups[32] at another NASA site in Santa Barbara, California, Remote Sensing Systems (hereafter referred to as RSS) took exception to the manner in which UAH team calibrated the data from ensuing satellites. Since the 25-year record came successively from nine such satellites, they all had to be put on the same absolute scale. Given the assumptions UAH made, most of these linkages between overlapping satellites seemed acceptable. But one in the 1991-1992 time frame looked suspicious, as it required a tenfold larger calibration factor than all the others. Note here that this is just the time frame that I highlighted in CFK03, suggesting that something strange had happened. I pointed out that the surface and satellite records agreed rather well from 1979 to 1991, and from 1992 to 2000 but that during 1991 the satellite record had suddenly stepwise dropped by about 0.2°K below the surface record. Because that was just when the atmosphere was reacting to the large injection of dust into the stratosphere by the eruption of Mt. Pinatubo, I wondered if this had not been the cause. Now there seemed evidence for an error in cross calibration of two satellites that was masked by the volcano-induced cooling. The RSS group revised this calibration and made other improvements, and the result was that the satellite record showed warming similar to that at the surface. UAH countered that these corrections were a matter of judgment and that, because their results were corroborated by the radiosondes that make *in-situ* measurements of temperature in the middle troposphere, theirs was the more correct reduction and RSS had made some errors.

However, evidence for a larger warming trend continued to build. Another group[53] reduced the satellite data by an independent method that corrected for both instrumental calibration errors and satellite orbital drift. This work found even more warming at altitude than that of the RSS group had.

At this point there appeared extremely important papers by Fu and Johanson and colleagues[17,18,16], which questioned another reduction procedure of the UAH group. Satellites measure temperature with several sensors or Multi-Sensing Units (MSUs). Two of them (MSU2 and MSU4) are most commonly used to determine trends. MSU2 measures temperature mostly in the troposphere but unfortunately also includes a 17% contribution from the lower stratosphere, which must be removed. To do this both UAH and RSS used a method that had the satellite look sideways towards the horizon in successive steps and then, with a theoretical model of the standard atmosphere, remove the stratospheric component. The resulting temperature record is referred to as T2LT (temperature from MSU2, lower troposphere). This work was critical of that approach and proposed a more accurate method, taking advantage of MSU4, which measures temperature in the stratosphere. Thus the two records overlap in the lower stratosphere and by their method determined another version of TLT (effectively slightly higher in the troposphere than the others). They then applied this method to both the UAH and RSS determinations. The result was that both now showed even greater warming.

It has been argued that this method is too inaccurate and unconstrained to be used. But in an elegant study[19], Gillett et al. used a climate model as a proxy for the actual atmosphere and applied the method to it. They found it gave extremely accurate results. To test this numerical method the model does not have to be perfect. It just has to produce the general characteristics of a troposphere with a cool stratosphere above it. We take from this that the method of combining sensors MSU2 and MSU4 is robust in separating out the stratospheric component in MSU2 and should be the preferred one to use.

Still another study seemed to support the RSS results. Santer et al.[42] showed that the altitude where the troposphere (cooling with altitude) met the stratosphere (warming with altitude), the "tropopause," had increased over the past quarter century and that computer models, which reproduced tropospheric warming and stratosphere cooling trends over that time period, predicted just the observed tropopause altitude change. Still the seeming agreement between satellite and radiosonde results—little warming trend—remained as a large boulder blocking the road to the anthropogenic greenhouse gas (AGHG) hypothesis.

This all changed in the fall of 2005 when three papers[33,44,49] appeared in a single issue of *Science* magazine. These three articles plus a fourth described below, taken together, essentially ended the problem of middle troposphere temperature trends' not agreeing with those measured at the earth's surface. In summary, Mears and Wentz[33] showed the effect of correcting for satellite drifts, which built on earlier work and further rendered MSU trends commensurate with surface trends. Santer et al.[44] showed that radiosonde-determined trends and lapse rates disagree with theory and models, which predict amplification of warming with altitude. Sherwood et al.[49] gave a strong argument for why the radiosondes are inaccurate. The resulting picture is that:

a. Satellite-observed warming is commensurate with that at the surface.
b. Thus the last quarter century has indeed seen dramatic warming.
c. Computer models appear to simulate this warming rather well in addition to agreeing with theory in warming amplification with altitude.
d. Given these results, it is a robust conclusion that the last quarter century has seen unprecedented warming that can be simulated only by adding AGHG forcing.

### a. Mears and Wentz[33]

This paper shows the effect of satellite drift such that meridian crossing had heretofore been inaccurately timed, which led to drifts in daily temperature retrievals. Application of their correction leads to warming in the tropics "consistent with that found at the surface."

Drifts of different satellites over the 25-year period of observation differ markedly. The main culprit seems to have been NOAA-11, which drifted more than 6 hours before its failure in 1998 (only its records from 1988-1993 were used here). This corresponds to the time of the eruption of volcano Mount Pinatubo in 1991. As mentioned above, I had noticed that the MSU measurement (UAH) of the subsequent cooling from its stratospheric aerosols was some 0.2°K more than observed at the surface. Prior to that, surface and MSU observations were in good agreement. Following that time they were also in fair agreement but with that 0.2°K offset. The UAH reductions apparently failed to properly account for the drift of NOAA-11 leading to a result of little or no warming. There was a similar drift problem mainly over the tropics in the post 1998 El Niño era.

The UAH trend during 1979-2005 was 0.087°K/decade. Correction for satellite drift led to an RSS-determined trend of 0.193°K/decade very similar to that determined at the surface. I note here that the UAH extra-tropical NH trend agrees fairly well with that at the surface. It is the over the tropics where UAH and surface records disagreed. Here the UAH trend was one of: cooling -0.015°K/decade which upon correction for satellite drift rose to +0.189°K/decade.

The authors went on to point out that these results agreed with those obtained by Fu and Johanson[18], discussed above. They also said that warming in the tropics is corroborated by observations of total column water vapor, a good indication of increasing temperature[51].

### b. Santer et al.[44]

This paper looks at what it sees as the physical impossibility of the UAH TLT trend being so different from that of the surface. Here some have argued that this is proof that the surface record is in error, but much detailed work has gone into substantiating the surface observations and, though they have problems, these are in total too small to account for the large difference in trends.

To address this point, this paper examines primarily the theory of wet adiabatic lapse rate, where moist rising air condenses, depositing its latent heat at ever-higher altitudes. This effect is itself a function of temperature such that heating at altitude should normally be greater than at the surface by up to 50-60%. The authors' thus inter-compare measured temperature changes with altitude with theory and with computer climate simulations over the observational time period. The time period for comparison was 1979-1999 where 1999 was the end of the IPCC historical forcing experiment, which the models were run for.

Now, everyone agrees that the stratosphere has cooled substantially. Comparisons over the tropics show that models and both UAH and RSS cooling trends for the stratosphere are similar, while radiosondes show even more cooling. Comparisons of the T2 trend (temperature in the troposphere determined from MSU2, which unfortunately includes some 17% contribution from the cooling lower stratosphere and thus is expected not to show a large a warming trend as if it measured only in the troposphere) from different sources show the following:

- radiosondes—cooling,
- UAH—slight warming,
- RSS—larger warming, and
- models even more.

Fu and Johanson's[18] stratospheric removal method showed increased warming for all observers in roughly the ratio as for T2. In the T2LT comparisons models show similar high levels of warming (~0.2°K/decade—recall the RSS result from Mears and Wentz[33] of 0.193°K/decade) while radiosondes show warming of ~0.05°K/decade.

These comparisons seem to support the theory of warming amplification with altitude excepting for UAH and the radiosondes. But the authors then looked for a possible reason for this by comparing the amplification in two different time scales—monthly and decadal averages. Here an interesting result is obtained which is telling. On monthly time scales amplification ratios plotted vs. height (in pressure units) show models, theory and sondes all agreeing rather well from the surface to nearly 300 mb (where the theory does not apply as well due to lack of uniform deep convection). But for decadal scales the sondes show marked disagreement with models and theory everywhere. It looks like something is wrong with radiosonde determination of long-term trends. The authors go on to speculate why the radiosondes could agree with RSS observations, models, and theory over short time scales but not over long ones. They suggest an answer that is dealt with convincingly by Sherwood et al.[49].

### c. Sherwood et al.[49]

Here, the authors note, "the temperature difference between adjacent 0000 and 1200 UTC weather balloon (radiosonde) reports shows a pervasive tendency toward cooler daytime trends compared with nighttime since the 1970s especially at tropical stations." They define a temperature difference which, averaged in longitude around the globe, should be near zero. A plot of this quantity over the tropics shows a large difference (~0.45°) in 1970, which subsequently reduces gradually to zero after 1997. They point out that the decadal trend with respect to altitude in this quantity is "almost two orders of magnitude larger than can be justified physically based on known forcings." Other similar inconsistencies are discussed.

They then "drop the bomb" as it were by suggesting that the trend difference between day and night was caused by changes in detector insulation against direct sunlight. Indeed radiosondes had been reporting a disquieting warming trend, but only at night when the sun was not shining directly on the detector. The authors discuss how this fits the observations from stratosphere to mid troposphere and also with latitude. Using MSU (satellite) data itself—diurnal-mean MSU trends between sites from the UAH reduction—they are able to quantify the temperature corrections that should be made to radiosonde data. The resulting tropical warming trend using this correction is 0.14°K/decade over the period 1979-1997. This is not as high as trend determinations above, but is certainly close enough to cease giving support to the UAH trend results. The authors end by discussing other problems with using radiosondes to get accurate multi-decadal trend determinations. Note here that this explanation also explains rather well the problem noted by Santer et al.[44], where radiosondes agreed with RSS observations, theory, and models on daily and monthly time frames but not over decadal periods.

If this were not sufficient to show that UAH and radiosonde results are incorrect, a fourth paper by Randel and Wu[40] showed similar problems with determining long-term temperature trends using radiosondes and came to essentially the same conclusions.

These papers added so much to the discussion that the U.S. Climate Change Climate Program published a rather large and comprehensive report[23] on what was known so far about the earth's temperature especially in the middle troposphere. It concluded that we now have general agreement among all data sets. A lingering problem, however, is that theory predicts more warming aloft than at the surface, which is not seen in all data sets. In the overall context of AGHG hypothesis, this is a second-order issue.

### NATURAL CLIMATE VARIABILITY

### a: Advances in determining climate change in the past 1,000 years

In CFK03, I detailed several attempts to determine global and in particular Northern Hemisphere (NH) average temperatures over the past 1,000 years or so. Perhaps the most well known of these is due to Mann et al.[30], popularly known as the "hockey stick" graph. While critics have attacked this work widely, they usually fail to note that some seven other similar determinations agree fairly well with it. However, the hockey stick does seem to have one significant deficiency—it misses the low-frequency variations, in particular the LIA. There were two of these: around the mid 1600s and the mid 1800s. (Recall that in CFK03 I noted that inversion of temperature measurements made in deep boreholes seemed to capture low-frequency variations and indicated the LIA was cooler than most of the above proxy determinations.)

The argument about Mann's hockey stick graph centers on two quantities, how cool it got during the LIA and how warm it got (compared with recent temperatures) during the MWP. The first of these has little direct relevance to present warmth except that it helps determine climate sensitivity to changes in solar activity. A colder LIA means that the climate responds more sensitively to changes in solar forcing. However, if the MWP was as warm as the present day northern hemisphere, one can argue our current climate is within bounds of natural variability, so that is an important consideration.

Three recent studies[54,55,34] have been cited by critics as showing that the hockey stick is in error and thereby questioning the source of current warmth. In reality, if carefully read, these three papers actually give strong support to the AGHG hypothesis. Von Storch et al.[54,55] use a climate model from the Max Plank Insititute, ECHO-G, to reconstruct temperatures over the past 1,000 years. The model result is subjected to differing amounts of numerical noise and a synthetic proxy temperature reconstruction is made for varying amounts of noise. The results show that, if subjected to the type and amount of noise as involved in the hockey stick proxies, the depth of the LIA is greatly reduced and agrees well with the hockey stick result when the actual answer is that the LIA was significantly cooler than Mann et al.[30] said it was. The modeled coolness of the LIA agrees more closely with that of bore holes (see discussion in CFK03).

The second study[34] addresses another problem besetting hockey-stick-type reconstructions. These seem to capture the high-frequency (annual to decadal) variability of climate rather well, but may miss the low-frequency (multi-decadal) swings. This paper adds low-frequency proxy data from oceans as well as land records. Other work used land records only to reconstruct NH average temperatures over the past 2,000 years. To combine low- and high-frequency data the authors use wavelet theory, a potentially powerful way to treat such data. This result shows a much cooler LIA than the hockey stick, more in agreement with borehole results. It also compares very well with von Storch's ECHO-G modeling results. Here we note that in order to get models to agree with the hockey stick and similar low-amplitude results, the climate sensitivity introduced must be lower than that determined from modeling 20th century temperatures. This was unsettling. Using a higher sensitivity (or a first principles model such as ECHO-G) makes the climate respond more strongly to variations in solar forcing and therefore gives a much cooler LIA. The good news is that the ECHO-G result, which agrees with the Moberg proxy reconstruction, yet has climate sensitivity similar to that for reproducing 20th century temperatures.

So far, so good, but what do these two efforts say about the MWP? Moberg's reconstruction essentially agrees with the many other higher frequency studies (including the Mann's hockey stick) in showing a NH average temperature about as warm as in the 1940s and significantly below current temperatures. However, with higher climate sensitivity one might expect the von Storch's ECHO-G model to respond to higher solar forcing with a warming MWP. In fact it does not. It continues to agree with all these proxy reconstructions. In a private communication, von Storch cautions that, since the ECHO-G runs were started just before the MWP, the model might not have "settled down"; however, there is no evidence that this is in fact a problem.

Osborn and Briffa[37] looked all this and other data and concluded: " Positive (*temperature*) anomalies during 890 to 1170 and negative anomalies during 1580 to 1850 are consistent with the concepts of a Medieval Warm Period and a Little Ice Age, but comparison with instrumental temperatures shows the spatial extent of recent warmth to be of greater significance than that during the medieval period." Another excellent short summary of this work is Bradley et al.[7].

Thus, despite considerable attacks on reconstruction of past climates, it appears that the most recent work, while showing the LIA to be cooler, as borehole data show it to be, the MWP was not as warm as at present, corroborating current estimates of the response of climate to high solar activity as being unable to explain current warmth.

While all this sounds fairly clear, the National Research Council's recent report to Congressional queries, "Surface Temperature Reconstructions for the Last 2,000 years"[26], took a more cautious stand saying that we cannot yet be highly certain just how warm the MWP was, indeed, that it might have been as warm as the last decade. The report, however, did say there is no evidence for a warmer MWP, just not enough proxies to give high confidence in any result. See also Kerr[27].

However, I think there is an additional observation that at least increases our certainty. This is Lonnie Thompson's experience with coring high mountain glaciers in the tropics[50]. Many of his cores go back tens of thousands of years or more. In some cases, when he attempted to re-core several of these some 20 years after he made the first cores, he had a hard time because the mountain glaciers had melted so extensively that it was hard to find a place to drill. Given the rapid pace of this melting, it is likely that many if not all of these mountain glaciers may be totally melted in the next decades. If this is so, then one might argue that the MWP could not have been as warm as at present—at least not in the tropics—else those glaciers would have melted to bedrock then, and Lonnie's cores would have found only ice that formed after the MWP. Thus the existence of mountain glacier cores going back tens of thousands of years would seem to imply that they did not melt much during the MWP and thus it was not as warm as today.

### b. Solar Forcing of Recent Climate Change

Attempts to determine the sun's influence have centered around two different approaches:

1) Interpretation of satellite-observed total insolation (TSI)—satellite calibration.
2) Magnitude of solar forcing necessary to reproduce past climate change, especially changes in the past 1,000 yrs or so—that is, paleo-calibration.

The two are not totally independent since those working on #2 use the calibrations from #1. But method #2 has the advantage (among many disadvantages) of trying out the calibrations from #1 on past climate, thereby giving some insight into both climate sensitivity to forcings and whether direct TSI is the dominant solar effect or if some indirect effects are also important (i.e., if past temperature variations cannot be simulated with direct TSI changes only, then one might infer that indirect solar forcings are at work also).

### 1) Satellite Calibration

Satellites have been observing TSI since 1979. This 27 yr record spans three solar maxima and nearly three minima. The maxima were roughly at 1980,1990, and 2001. The minima were about 6-7 years after these, and so we are approaching the third minimum, which should occur near 2007-8. These observations allow us to calibrate changes in solar activity determined over the past 1,000 yrs by proxies such as sunspot number or concentrations of the isotopes $^{10}$Be or $^{14}$C. With them it is possible to quantify direct solar forcing of climate (see extensive discussion in CFK03). Resulting estimates are that changes in solar activity in the past century have caused ¼ - ½ of the observed warming up to 1980, but since the solar maxima of 1990 and 2001 were no higher than that in 1980, the sun cannot account for the rapid warming in the past 26 years. However there is one objection to this conclusion, namely that, while there has been no increase in solar maxima, there may have been an increase in solar activity at *minimum*. If this were so, Scafetta and West[46,47], using models that incorporate this extra TSI forcing, have argued that solar activity might be responsible for perhaps half of the warming since 1980. However, this approach has its drawbacks even if the observed TSI at solar minima were real. An extensive critique of this paper is available at the web site Real Climate.

In addition there is disagreement among those who reduce satellite observations as to whether there has indeed been an increase in TSI at the 1996-7 minimum. Two reductions have been done. One by Willson and Mordvinov[57]shows an increase. The other by Frølich and Lean[14,15] shows none. The differing results occur because there is a time period where satellite coverage of TSI is poor and/or incomplete. There were different satellites involved during that crucial time and which answer one gets depends on which satellite observations are used. Willson[57] finds an increase. He (personal communication, 2006) points out that Lean's result depends on a sensor known to have changed in its sensitivity over time and therefore unreliable. However, Lean[28] points out that if you difference the two results they agree well to the point that there is a sudden stepwise jump in the Willson determination just at the 1996 minimum, which seems unphysical (changes in sensor sensitivity are generally uniform and gradual, not abrupt). And so I prefer Lean's "no increase" result. Perhaps this will all become moot as we are now observing a third minimum, which thus far looks like it will agree with Lean's "no increase" version.

### 2) Paleo-Calibration

Much effort has gone into using proxies (tree-rings, isotopes, ocean sediments, etc.) to determine northern hemisphere and global temperatures over the past millennium. The most widely known is Michael Mann's "hockey stick" result discussed previously, but it is not the only one. There are an increasing number of such determinations (see CFK03 and Section 2a this paper). The more recent ones disagree with Mann et al.[30] during the two cool periods of the LIA. They show them to be substantially cooler more in agreement with borehole measurements. But surprisingly they differ little from Mann's moderately warm MWP showing it to be only as warm at in the 1940's.

Computer models simulate temperatures in this time frame using the satellite-calibrated $^{10}Be$ and $^{14}C$ proxies for variations in solar activity. As discussed in Section 2a, current computer simulations with accepted levels of climate sensitivity to forcings (solar, volcanic, GHG, etc) seem to be reproducing climate in the past 1,000 yrs. rather well, thus agreeing with the proxy reductions that the MWP was only about a warm as in the 1940's. Since these models employ only direct TSI solar forcing, it would seem that other indirect solar effects (see CFK03), while possibly present, are not sufficiently strong to significantly drive climate change. This conclusion is largely substantiated by other recent papers[3,13].

### MODELS AND THEIR PREDICTIVE CAPABILITY

One of the cornerstones of the critics' position against the AGHG hypothesis is that models, although they can represent certain simple aspects of climate, are essentially incapable of simulating the real climate in all its complexities and therefore cannot be used for reliable predictions. In CFK03, I gave several examples of significant predictive capability from predicting the actual warming twenty years in advance to simulating accurate ocean warming in different ocean basins before the data corroborating it were known. Since 2002, models have become an everyday tool for parsing different physical aspects of climate dynamics, for understanding the meaning of data sets as well as for predicting future climate. Here I mention a few additional ways models have shown considerable predictive ability.

Perhaps the most dramatic prediction was that the middle troposphere was warming as much or more than the surface. For a decade models were attacked because reductions of satellite and radiosonde data (see Section 1) were showing no such warming. Thus, it was said that, if the models couldn't even get this fundamental aspect of climate correctly, how could we put faith in any of their more general predictions? Today we know that the data reductions were the ones in error and that the models were correct all along. In fact models were used over and over again in comparisons with those data sets to call them into question[41,43]. Other examples of model capability include their ability to reproduce past climate known from proxy data sets (Section 2a and b) and in particular their ability to quantify the magnitude of natural forcings such as volcanic and solar and their role in affecting climate change.

In an update of work reported on in CFK03, Hegerl and Bindoff[20] and Barnett et al.[4], have done more detailed study of recently observed warming in the vertical column of various global oceans[29]. In their abstract Barnett et al.[4]say: "A warming signal has penetrated into the world's oceans over the past 40 years. The signal is complex, with a vertical structure that varies widely by ocean; it cannot be explained by natural internal climate variability or solar and volcanic forcing, but is well simulated by two anthropogenically forced climate models. We conclude that it is of human origin, a conclusion robust to observational sampling and model differences. Changes in advection combine with surface forcing to give the overall warming pattern."

Their conclusions follow in which they make a larger point—models like these can indeed make useful predictions:

> "The implications of our results go far beyond identifying the reasons for ocean warming. First, they show that uncertainties in the models used here are too small to affect the conclusion attributing the historic ocean warming signal to anthropogenic forcings, at least for the temperature-driven part of the signal. Second, taking these new results with those obtained in the last few years [e.g.,25,42,45] leaves little doubt that there is a human-induced signal in the environment. Third, because the historical changes have been well simulated, future changes predicted by these global models are apt to be reasonably good, at least out to, say, 20 to 30 years into the future. How to respond to the serious problems posed by these predictions is a question that society must decide."

In passing I note that the rhetorical logic of many critics is a bit difficult to follow. They start out with a principal: "The climate is too complex and subject to too many different forcings and feedbacks for any computer model to capture them well enough to make predictions." Having dismissed the extremely complex computer models that consider the most important forcings and feedbacks globally and at each time step, they go on to ignore their own statement of complexity and give their own intuitions as to what the climate will do or is doing. Climate models do indeed still have significant deficiencies. Their horizontal resolution is still not sufficiently fine to allow regional predictions of more than the most general nature. Their treatment of cloudiness, water or ice content, etc. is still rudimentary enough to caution their quantification of cloud feedback processes (although they seem to be reproducing many aspects of clouds and cloud feedback fairly well). For example they still do not simulate stratiform clouds over the equatorial ocean off the coast of Peru or in the Arctic well, and the problem of their generating an unphysical double intertropical convergence zone in the western Pacific is still a matter of concern and study. **Author note added in proof**: recent new mini-models of deep convection in the climate models seem to be getting rid of the unphysical double ITCZ. Regardless, models have come into daily use in both understanding past climate and predicting the future. And they are increasingly being used to assist in planning future observational programs.

### SECOND ORDER, BUT IMPORTANT, CONSIDERATIONS

This last section will list and briefly discuss some facets of climate change that are growing in importance but which are not in the main line of the reasoning for how much humans are causing the change.

### a. Land Use

Humans have been altering the natural landscape for thousands of years, but mostly in the past few hundred. While such alterations generate a multifaceted climate response there are roughly two major impacts. Cultivation of dry lands on balance decreases albedo (the ratio of sunlight reflected from a surface to that incident, where albedo of 1 is complete reflection and 0 is complete absorption), because more land is covered with light-absorbing plants. Countering this is the increase in albedo caused by clearing of highly absorptive rain-forest vegetation. The total effect is thought to be small if only because land surface is small compared with ocean area. Nevertheless, recent studies[38,12] are making the point that we need to get the best estimates of this effect possible to more certain of its affect on climate change.

Further, land changes are being shown to have secondary effects on moisture content of the atmosphere, which also need to be taken into consideration. Although important, in a global sense such effects are most probably second order because much of the land use change has already occurred. Regardless, continued deforestation in the tropics and other continuing changes need to be monitored and included in modeling efforts. Within a comprehensive report on all climate forcings is a good summary of the land use question[35].

One place where land use does have a noticeable effect is in regional changes. Here, while not necessarily contributing to global warming, local populations might see their regional climate undergoing quite noticeable changes such as those documented in Florida in the past century[31].

### b. Aerosols and Clouds: Global Dimming

A more important aspect of climate forcing is the subject of particulates suspended in the troposphere. While these have resident times of only a few weeks, human activities are constantly replenishing them. Major components are solar-reflecting sulfates and solar-absorbing black carbon soot. It has long been understood that such air pollution has a net cooling effect on recent climate. Indeed, in the 1950s and 60s it was large enough to obscure the AGHG warming.

This is because in addition to their direct forcing, particulates affect cloud water content and indeed cloud amounts. They do this mainly by adding cloud condensation nuclei to the atmosphere causing clouds to have a preponderance of small droplets, which increases the ability of clouds to scatter solar radiation rendering the clouds "brighter" and thus increasing local albedo (more reflection of sunlight back into space), thereby increasing the net cooling effect. This indirect effect has been difficult to quantify and has been treated as somewhat of a limited but bounded free parameter in climate simulation codes. Satellite data and laboratory study are beginning to quantify this effect, and it is seen that in some cases the net cooling caused by heavy concentrations can still nearly cancel the warming effect of AGHGs[6,24].

At the other end of the spectrum, black carbon soot absorbs incoming solar radiation and warms the atmosphere at the altitude of the soot layers. In some cases this decreases the local albedo, contributing to warming, but not in all. Instead, it simply changes where in the troposphere sunlight is absorbed. However this can have dramatic effects on clouds and rainfall because it changes the vertical heating in the atmosphere cooling the surface and warming the higher regions.

Nowhere has this effect been better observed than in the Indian Ocean as reported by Ramanathan and colleagues[39,10]. In a large multi-year observational exercise called INDOEX, they documented large "brown cloud" pollution moving south from the Indian subcontinent. This caused global dimming and the northern Indian Ocean cooled, thus reducing evaporation and consequent monsoon rains by some 10% since the 1950s. Meanwhile the southern Indian Ocean has continued to warm with the rest of the planet resulting in increased rainfall in parts of the Sahel in Africa. Such absorbing aerosols occur elsewhere in the world and most likely are having similar affects on regional climate

Another way to look at this effect is to measure the total incoming solar radiation at different sites around the northern hemisphere. These measurements, carried out over the past 40-50 years show what has been termed "global dimming" in that there was less sunlight reaching the earth's surface in 1990 than in the past[1]. It is thought that this phenomenon is largely due to particulates' both direct and indirect effects. However, more recent measurements[56] show the dimming diminishing since that time. In the abstract of that paper the authors state:

> "A decline in solar radiation at land surfaces has become apparent in many observational records up to 1990, a phenomenon known as global dimming. Newly available surface observations from 1990 to the present, primarily from the Northern Hemisphere, show that the dimming did not persist into the 1990s. Instead, a widespread brightening has been observed since the late 1980s. This reversal is reconcilable with changes in cloudiness and atmospheric transmission and may substantially affect surface climate, the hydrological cycle, glaciers, and ecosystems."

They conclude on a cautionary note that, as the aerosol-induced dimming decreases, the full effects of AGHG warming will begin to be felt.

### c. Glaciers—Greenland, Antarctica and in the Tropics

Glaciers the worlds around are in retreat. What does that mean? Critics say that it is just their continuing response to early 20th century warming as the Earth emerged from the last LIA. They also say glaciers can retreat not only due to melting but as a consequence of reduced precipitation. Greenland and Antarctica are the places where people worry most about melting, because they hold the largest amount of ice and can raise sea level to extremely high levels. Currently the behavior of their glaciers is mixed. Parts of both Greenland and Antarctica have glaciers that are moving rapidly towards the sea and as a consequence contributing to sea level rise. But other parts are not doing this. The real question to ask here is whether retreat of mountain or rapid movement of sea level glaciers is slowing or accelerating. If in response to first half of 20th century warming, they should be at least not accelerating if not actually slowing. On the other hand, if in response to late 20th and early 21st century continued warming, they should be accelerating their respective melt or movement towards the sea.

There is increasing evidence for acceleration. Greenland has not been warming much until just recently largely due to persistent weather patterns that allow Arctic air to move south over it. However, the most recent satellite and surface measurements are starting to show accelerated melting and more rapid movement[26, and several papers in that issue of *Science*). It is thought that melt water is penetrating to the base of the glaciers lubricating them and causing them to move faster. However, here one needs to look at the ability of the mid-range snowfields to feed these racing glaciers. While lubricated glaciers can indeed move what ice is in the "pipeline" to the sea rapidly, the ice fields above that feed them show little sign of increasing the amount of ice they feed into the glacial channels. So how much of a threat these glaciers pose to sea level rise will have to be watched.

Antarctica is another story (Alley et al.[2]). About half of it is warming and ice is both melting and accelerating its movement to the sea. The other half is actually increasing the amount of ice "stored" on land. Two recent papers draw different conclusions. Alley in a review article says Antarctica is nearly in ice balance because east ice increasing due to increased snowfall and west decreasing due to rapid glacier movement. But a more recent paper in *Science Express*[52] claims that time-variable gravity measurements show mass loss since 2002. Exacerbating the melting is the breakup of the huge areas of floating ice packs. These have been frozen solid well back in time but are now broken up. Solid floating ice packs retard the flow of land glaciers into the sea, but this is no longer the case, and many glaciers are moving large amounts of ice into the ocean. However, again if this rapid pace can keep up since the ice fields above them seem to feed at a steady rate which is slower than current down stream movement. It appears that melting sea level glaciers are not contributing a large amount to sea level rise yet, but, if warming continues, they could do so with increasing rapidity towards the end of this century and thereafter.

Another way to look at past climate change is to study mountain glaciers from the highest peaks in the tropics. Lonnie Thompson and his team have been doing this for several decades. It is quite a story since they much not only climb the peaks carrying tons of ice coring equipment, but also must remain there for weeks drilling the cores. (For a readable telling of this story and what they have found out see Bowen, 2005). They found to their surprise that these glaciers held a climate record, not just a few hundred years old, but mostly over 10,000 years old. This work is particularly important since most ice core studies have been limited to Polar Regions that might not be representative of climate change over most of the earth. These were records from the tropics, which many think is the place most of the Earth's climate comes from. One of the most interesting discoveries was that these glaciers help solve what has been called the "Mercer Problem" named for its discoverer, namely that Antarctica ice cores show the last ice age gradually ending some 2,000-3,000 years before it did in Greenland. Thus it would seem that the end of the ice age began in the southern hemisphere. Thompson's mountain glaciers generally agree with the Antarctic results[50].

This caused another team[48] to look more closely at glacial activity in the northern subtropics such as the United States. Working with geologists who can date the retreat of North American ice sheets, they were able to find that these also began retreating about 17,500 years ago. The picture now emerging is one that seems to isolate Greenland as one of the only places where warming did not start until 15,000 years ago. As a side note, this and other findings are showing increasingly that Greenland is often not representative of climate elsewhere around the globe. Thompson's team recently published a summary of their work in the Andes, Africa, and the Himalayas. While temperature records from different locations often differ substantially with time, their composite average is in remarkable agreement with other attempts to use proxies to determine temperatures in the past few thousand years

(Section 2A this review) and adds yet another datum to show that the Medieval Warming Period was not as warm as today.

### d. Sea Level Rise

Sea levels have been rising since the end of the beginning of the Holocene some 10,000 yrs ago. The present century long warming has seen a steady of rise of some 2 mm/yr as a result of warming since the last glacial period, but this rate seems to have increased recently by about 60%. Global changes in ocean levels (not all are rising due to various factors) are being closely watched by the Topex-Poseidon Satellite program, which accurately measures sea-level height. Most of the rise is due to expansion of the warming ocean with an increasing contribution from melting glacial ice. It seems certain that the rate of sea level rise will continue to increase, but by how much is uncertain. Predictions remain about as they were in CFK03—about half a meter per century. Perhaps the most recent work on this subject is by Church and White[11]. Their abstract follows, which after a large very inclusive effort, essentially supports the last IPCC's estimate of possible sea-level rise by 2100:

> "Multi-century sea-level records and climate models indicate an acceleration of sea-level rise, but no 20th century acceleration has previously been detected. A reconstruction of global sea level using tide-gauge data from 1950 to 2000 indicates a larger rate of rise after 1993 and other periods of rapid sea-level rise but no significant acceleration over this period. Here, we extend the reconstruction of global mean sea level back to 1870 and find a sea-level rise from January 1870 to December 2004 of 195 mm, a 20th century rate of sea-level rise of 1.7 ± 0.3 mm /yr and a significant acceleration of sea-level rise of 0.013 ± 0.006 mm /yr. This acceleration is an important confirmation of climate change simulations, which show an acceleration not previously observed. If this acceleration remained constant then the 1990 to 2100 rise would range from 280 to 340 mm, consistent with projections in the IPCC TAR."

### CONCLUSIONS

These past few years have been momentous ones, because they have seen research that pretty much solves the remaining problems that confronted the AGHG hypothesis. In the CFK03, I wrote that for all the complexity of climate and climate change we really only needed to ask a few questions:

1. Was the warming real? if so
2. Was it beyond natural variation? if so
3. Could we understand what was causing it, and could we predict what might happen in future scenarios?

This update has attempted to show that all these questions have now been answered in the affirmative. With the solution to the satellite/radiosonde vs. surface trend conundrum we can be confident that wherever temperatures are measured (including in the oceans), the warming is indeed real. While we still do not have total confidence in whether the MWP was as warm as the 1990s, there is a growing mass of evidence from various sources that says it wasn't.

Finally, I guess it is time to say it: "Don't bet against the computer simulations". They need constant improvement and still have important deficiencies, but these are to my mind second order ones—important, but not major. The models have passed too many tests and are in constant use in sorting out many climate problems. And their predictive capability seems well enough established that we can no longer disregard the picture they paint of continued global warming.

In fact, if the last 15 or so years of research are looked at, what is seen is a constant working out of the problems associated with the AGHG hypothesis and the systematic construction of an understanding of how the climate system works. Already research groups are beginning to incorporate into their planning studies aimed at evaluating not whether humans are warming the climate but rather what are the best approaches to slowing and indeed stopping that warming. It has been a remarkable example of concerted international efforts concentrated on one of the great problems facing us, and it appears it is being successful. There will still be problems and surprises, but these last few years have seen the end of the beginning.

### AUTHOR’S NOTE ADDED IN PROOF

Since this update was written, the IPCC issued its Summary for Policymakers of the scientific basis volume of the forthcoming 4AR on the state of knowledge about AGHG warming.

The summary makes some significant changes in the certainty with which scientists know what is happening and what is likely to happen in future. Some of these are quoted below from that report. While this report deals specifically with some of the more hotly contested issues considered in this update, it does so in a rather abstract and formal way. Thus, while it provides raw material for thinking about these issues, it does not of itself make it easy to confront critics and scoffers. In particular, the summary does not say how we know these things or what caused our certainty to increase. For this information one would have to plow through the much larger main text of this review., which is expected by mid 2007. One of the purposes of CFK03 and this update has been to provide a halfway point between mere assertion and details of the basis for those assertions. This is perhaps the difference between the present update and the more scholarly and in-depth approach of the IPCC report. Given the questions I am constantly asked—how to respond to certain critics' statements—I believe there is need for both kinds of treatments. Also in this update, I have not given any new prognostications of what climate change might be expected in future depending on how the world society decides to limit its GHG emissions. The IPCC Summary does this quite well, and I recommend reviewing it if you want the most up-to-date information on this issue. Finally, it is quite instructive to look at the Figure SPM-6 of that summary: "Projected Patterns of Precipitation Changes" between the two periods 1980-1999 and 2090-2099. It shows increases in precipitation in the tropics and at high latitudes with sometimes-major decreases in the mid-latitudes—food for thought.

Below are some selected statements from the 4AR Summary for Policymakers. Note that "TAR" refers to the previous IPCC Report, the Third Assessment Report, which was released in 2001 (Houghton, et al., 2001).

- Eleven of the last twelve years (1995-2006) rank among the 12 warmest years in the instrumental record of global surface temperature (since 1850). The updated 100-year linear trend (1906-2005) of 0.74 [0.56 to 0.92]°C is therefore larger than the corresponding trend for 1901-2000 given in the TAR of 0.6 [0.4 to 0.8]°C. The linear warming trend over the last 50 years (0.13 [0.10 to 0.16]°C per decade) is nearly twice that for the last 100 years. The total temperature increase from 1850-1899 to 2001-2005 is 0.76 [0.57 to 0.95]°C. Urban heat island effects are real but local, and have a negligible influence (less than 0.006°C per decade over land and zero over the oceans) on these values.
- New analyses of balloon-borne and satellite measurements of lower- and mid-tropospheric temperatures show warming rates that are similar to those of the surface temperature record and are consistent within their respective uncertainties, largely reconciling a discrepancy noted in the TAR.
- The average atmospheric water vapor content has increased since at least the 1980s over land and ocean as well as in the upper troposphere. The increase is broadly consistent with the extra water vapor that warmer air can hold.
- Observations since 1961 show that the average temperature of the global ocean has increased to depths of at least 3000 m and that the ocean has been absorbing more than 80% of the heat added to the climate system. Such warming causes seawater to expand, contributing to sea level rise.

- Mountain glaciers and snow cover have declined on average in both hemispheres. Widespread decreases in glaciers and ice caps have contributed to sea level rise (ice caps do not include contributions from the Greenland and Antarctic ice sheets).
- New data since the TAR now show that losses from the ice sheets of Greenland and Antarctica have very likely contributed to sea level rise over 1993 to 2003. Flow speed has increased for some Greenland and Antarctic outlet glaciers, which drain ice from the interior of the ice sheets. The corresponding increased ice sheet mass loss has often followed thinning, reduction or loss of ice shelves or loss of floating glacier tongues. Such dynamical ice loss is sufficient to explain most of the Antarctic net mass loss and approximately half of the Greenland net mass loss. The remainder of the ice loss from Greenland has occurred because losses due to melting have exceeded accumulation due to snowfall.
- Global average sea level rose at an average rate of 1.8 [1.3 to 2.3] mm per year over 1961 to 2003. The rate was faster over 1993 to 2003, about 3.1 [2.4 to 3.8] mm per year. Whether the faster rate for 1993 to 2003 reflects decadal variability or an increase in the longer-term trend is unclear. There is high confidence that the rate of observed sea level rise increased from the 19th to the 20th century. The total 20th century rise is estimated to be 0.17 [0.12 to 0.22] m.
- For 1993-2003, the sum of the climate contributions is consistent within uncertainties with the total sea level rise that is directly observed. These estimates are based on improved satellite and in-situ data now available. For the period of 1961 to 2003, the sum of climate contributions is estimated to be smaller than the observed sea level rise. The TAR reported a similar discrepancy for 1910 to 1990.

At continental, regional, and ocean basin scales, numerous long-term changes in climate have been observed. These include changes in Arctic temperatures and ice, widespread changes in precipitation amounts, ocean salinity, wind patterns and aspects of extreme weather including droughts, heavy precipitation, heat waves and the intensity of tropical cyclones.

- Average Arctic temperatures increased at almost twice the global average rate in the past 100 years. Arctic temperatures have high decadal variability, and a warm period was also observed from 1925 to 1945.
- Satellite data since 1978 show that annual average Arctic sea ice extent has shrunk by 2.7 [2.1 to 3.3]% per decade, with larger decreases in summer of 7.4 [5.0 to 9.8]% per decade. These values are consistent with those reported in the TAR.
- Temperatures at the top of the permafrost layer have generally increased since the 1980s in the Arctic (by up to 3°C). The maximum area covered by seasonally frozen ground has decreased by about 7% in the Northern Hemisphere since 1900, with a decrease in spring of up to 15%.
- Long-term trends from 1900 to 2005 have been observed in precipitation amount over many large regions. Significantly increased precipitation has been observed in eastern parts of North and South America, northern Europe and northern and central Asia. Drying has been observed in the Sahel, the Mediterranean, southern Africa and parts of southern Asia. Precipitation is highly variable spatially and temporally, and data are limited in some regions. Long-term trends have not been observed for the other large regions assessed.
- Changes in precipitation and evaporation over the oceans are suggested by freshening of mid and high latitude waters together with increased salinity in low latitude waters.
- Mid-latitude westerly winds have strengthened in both hemispheres since the 1960s.
- More intense and longer droughts have been observed over wider areas since the 1970s, particularly in the tropics and subtropics. Increased drying linked with higher temperatures and decreased precipitation has contributed to changes in drought. Changes in sea surface temperatures (SST), wind patterns, and decreased snow pack and snow cover have also been linked to droughts.

- The frequency of heavy precipitation events has increased over most land areas, consistent with warming and observed increases of atmospheric water vapor.
- Widespread changes in extreme temperatures have been observed over the last 50 years. Cold days, cold nights and frost have become less frequent, while hot days, hot nights, and heat waves have become more frequent.
- There is observational evidence for an increase of intense tropical cyclone activity in the North Atlantic since about 1970, correlated with increases of tropical sea surface temperatures. There are also suggestions of increased intense tropical cyclone activity in some other regions where concerns over data quality are greater. Multi-decadal variability and the quality of the tropical cyclone records prior to routine satellite observations in about 1970 complicate the detection of long-term trends in tropical cyclone activity. There is no clear trend in the annual numbers of tropical cyclones.

Some aspects of climate have not been observed to change.

- A decrease in diurnal temperature range (DTR) was reported in the TAR, but the data available then extended only from 1950 to 1993. Updated observations reveal that DTR has not changed from 1979 to 2004 as both day- and night-time temperature have risen at about the same rate. The trends are highly variable from one region to another.
- Antarctic sea ice extent continues to show inter-annual variability and localized changes but no statistically significant average trends, consistent with the lack of warming reflected in atmospheric temperatures averaged across the region.
- There is insufficient evidence to determine whether trends exist in the meridional overturning circulation of the global ocean or in small-scale phenomena such as tornadoes, hail, lightning and dust storms.

Paleoclimate information supports the interpretation that the warmth of the last half-century is unusual in at least the previous 1300 years. The last time the Polar Regions were significantly warmer than present for an extended period (about 125,000 years ago), reductions in polar ice volume led to 4 to 6 meters of sea level rise.

It is very unlikely that climate changes of at least the seven centuries prior to 1950 were due to variability generated within the climate system alone. A significant fraction of the reconstructed Northern Hemisphere interdecadal temperature variability over those centuries is very likely attributable to volcanic eruptions and changes in solar irradiance, and it is likely that anthropogenic forcing contributed to the early 20th century warming evident in these records.

### ACKNOWLEDGEMENTS

This work was made possible by Los Alamos National Laboratory's Branch of the University of California's Institute of Geophysics and Planetary Physics, I would like to thank its Director, Dr. Gary Geernaert and his administrative aide, Deb Rivera for their assistance and encouragement. Also I thank my wife for her critical editing and interest. Thanks are also due to the many researchers who answered my questions on email: Michael MacCracken, Richard Alley, Judith Lean, Richard Willson, Hans von Storch, Ben Santer, Qiang Fu, Roy Spencer, Roger Pielke Sr., Lonnie Thompson, Phil Jones and Mathew Hecht. The Los Alamos National Laboratory is operated by the U.S. Department of Energy by the University of California. The views expressed here are those of the author and do not necessarily reflect the views of these organizations.

### REFERENCES

1. Abakumova, G.M., Feigelson, E.M., and Stadnik, V.V. (2001) Global dimming: a review of the evidence for a widespread and significant reduction in global radiation with discussion of its probable causes and possible agricultural consequences, **107**, 255-278.
2. Alley, R.B.Clark, R.U., Huybrechts, and P. Joughin, I. (2006) Ice-Sheet and Sea-Level Changes *Science*, **310**, 456-460.
3. Barda E., and Martin F. (2006) Climate change and solar variability: What's new under the sun?, *Earth and Planetary Science Letters*, **248,** 1-1432.
4. Barnett et al. (2005) Penetration of human-Induced warming into the world's oceans, *Science*, **309**, 284-287.
5. Bowen, M. (2005) Thin Ice: Unlocking the secrets of climate in the world's highest mountains" ISBN-13:9788-0-800-643-8, ISBN-10: 0-8050-6443-5.
6. Breon, F.M. (2006) Perspectives: How Do Aerosols Affect Cloudiness and Climate? *Science*, **313,** 623-24
7. Bradley, R.S., Hughes, M.K., and Diaz, H.F., (2003) Climate in Medieval Time, *Science*, **302**, 404-405.
8. Christy, J.R., Spencer, R.W., and Lobl, E. (1998) Analysis of the merging procedure for the MSU daily temperature time series, *J. Climate*, **11**, 2016-2041.
9. Christy, J.R., Spencer, R.W., Norris, W.B., Braswell, W.D., and Parker, D.E., (2003) Error estimates of Version 5.0 of MSU/AMSU bulk atmospheric temperatures *J. Atos. Ocean. Tech.* **20**, 613.
10. Chung, C.E., and Ramanathan, V. (2006) Weakening of N. Indian SST gradients and the monsoon rainfall in India and the Sahel, *J. Climate*, **19**, 2036-2045.
11. Church, J.A., and White N.J. (2006) A 20th Century global sea-level rise, *Geophys. Resear.Lett.* **33**, L01602.
12. Fedema, et al. (2005) The Importance of Land-Cover change in simulating future climates, *Science*, **310**, 1674-1678.
13. Foukal, P., Frølich, C., Spruit, H., and Wigley, T.M.L. (2006) Variations in solar luminosity and their effect on the Earth's climate, **443**, 161-166.
14. Frølich, C., and Lean, J. (1998) Sun's total irradiance: Cycles, trends, and related climate change uncertainties since 1976, *Geophys. Res. Lett.*, **25**, 4377-4380.
15. Frølich, C., and Lean, J. (2004) Solar radiative output and its variability: Evidence and mechanisms, *Astron. Astrophys. Rev*. **12**, 4.
16. Fu, Q., and Johanson, M. (2004) Stratospheric influences on MSU-derived tropospheric temperature trends: A direct error analysis, *J. of Climate*, **17,** 4636-4640.
17. Fu, Q., Johanson, M., Warren, S.F., and Seidel, D.J. (2004) Contribution of stratospheric cooling to satellite-inferred tropospheric temperature trends. *Nature*, **429,** 55-58.
18. Fu, Q., and Johanson, C.M., (2005) Satellite-derived vertical dependence of tropical tropospheric temperature trends *Geophys. Res. Lett.*, **32**, L10703
19. Gillett, N.P., Santer, B.D., and Weaver, A.J. (2004), Stratospheric cooling and the troposphere. *Nature*, published online (brief communications arising) 2 December 2004, (10.1038/nature 03208) 1-2.
20. Hegerl, G.C., and Bindoff, N.L. (2005) Warming the World's Oceans, *Science*, **309**, 254.
21. Houghton, J.T et al., Eds. (2001) IPCC, "WG1 Third Assessment Report".
22. IPCC (Intergovernmental Panel on Climate Change) (2007) http://ipcc-wg1.ucar.edu/wg1/docs/WG1AR4_SPM_PlenaryApproved.pdf
23. Karl, T.R. Hassol, S.J., Miller, C.D., and Murray, W.L., Eds. (2006) Temperature Trends in the Lower Atmosphere: Steps for Understanding and Reconciling Differences, Report by the U.S. Climate Change Science Program and the subcommittee on Global Change Research, 164. http://www.climatescience.gov/Library/sap/sap1-1/finalreport/sap1-1-final-all.pdf
24. Kaufman, J.J., and Koren, I. (2006) Smoke and pollution: aerosol effect on cloud cover, *Science*, **313,** 655-658.
25. Keller, C.F. (2003) Global warming: The balance of evidence and its policy implications. *TheScientificWorldJOURNAL* **3**, 357-411. http://www.thescientificworld.com/SCIENTIFICWORLDJOURNAL/toc/TSWJ_ArticleLanding.asp?jid=141&ArticleId=1240
26. Kerr, R.A. (2006a) A worrying trend of less ice, higher seas. *Science* **311,** 1698-1701.
27. Kerr, R. (2006b) Yes, It's been getting warmer in here since the CO2 began to rise, *Science*, **312**, 1854.
28. Lean, J. (2006) Comment on "Estimated solar contribution to the global surface warming using the ACRIM TSI satellite composite" by N. Scafetta and B.J. West, *Geophys. Res. Lett.*, **33,** L15701-03.
29. Levitus, S., Antonov, J., and Boyer, T., (2005), Warming of the world ocean, (1955-2003) *Geophys. Res. Lett.*, **32.** L02604.
30. Mann, M.E., Bradley, R.S., and Hughes, M.K. (1999) Northern hemisphere temperatures during the past millennium: Inferences, uncertainties, and limitations, *Geophys. Res. Lett.*, **26**, 759–762.
31. Marshall Jr., C.H., Pielke Sr., R.A., Steyaert, L.T., and Willard, D.A. (2004) The Impact of Anthropogenic Land-Cover change on the Florida Peninsula Sea breezes and warm season sensible weather, *Mon. Weather Rev*., **132**, 28.

32. Mears, C.Q., Schabel, M.C., and Wentz, F.J., (2003) A Reanalysis of the MSU ? Channel 2 Tropospheric /Temperature /Record, *J. Clim.* **16**, 3650.
33. Mears, C.A. and Wentz, F.J. (2005), The effect of diurnal correction on Satellite-derived lower tropospheric temperature, *Science*, **309**, 1548-1551.
34. Moberg, A., Sonechkin, d.M., Holmgren, K., Datsenki, N.M., and Karlen, W. (2005) Highly variable Northern Hemisphere temperatures reconstructed from low-and-high resolution proxy data, *Nature*, **433**, 613-617.
35. National Research Council--Board on Atmospheric Sciences and Climate, (2005) Radiative Forcing of Climate Change: Expanding the Concept and Addressing Uncertainties, http://www.nap.edu/openbook/0309095069/html
36. North, G.R. (2006)"Surface Temperture Reconstructions for the Last 2,000 years." NRC Statement to Subcommittee on Oversight and Investigations, Committee on Energy and Commerce, U.S. House of Representatives, http://www7.nationalacademies.org/ocga/testimony/Surface_Temperature_Reconstructions.asp
37. Osborn, T.J., and Briffa, K.R., (2006), The spatial extent of 20th -century warmth in the context of the past 1200 years, *Science*, **311,** 841-844.
38. Pielke Sr., R.A. (2005) Perspectives: Land use and climate change. *Science*, **310**, 1625-1626.
39. Ramanathan et al, (2005) Atmospheric brown clouds: Impacts on South Asian climate and hydrological cycle, Academy of Sciences
40. Randel, W.J. and Wu Fei, (2006), Biases in stratospheric and tropospheric temperature trends derived from historical radiosonde data, *J. Climate*, **19**, 2094-2104.
41. Santer. et al. (2000) Interpreting differential temperature trends at the surface and in the lower troposphere, *Science*, **287**, 1227-1232.
42. Santer et al. (2003) Contributions of anthropogenic and natural forcing to recent tropopause height changes, *Science* **301,** 479-483.
43. Santer et al., (2004), Identification of anthropogenic climate change using a second-generation reanalysis, *Jour. Geophys, Res.*, *Atmospheres*, **109**, doi:10.1029/204JD005075.
44. Santer et al., (2005) Amplification of surface temperature trends and variability in the tropical atmosphere, *Science*, **309**, 1551-56.
45. Santer, B.D., J. E. Penner, P. W. Thorne (2006) In Temperature Trends in the Lower Atmosphere: Steps for Understanding and Reconciling Differences (Report by the U.S. Climate Change Science Plan and the Subcommittee on Global Change Research, Washington, DC.
46. Scafetta, N., and West, B.J. (2005) Estimated solar contribution to the global surface warming using the ACRIM TSI satellite coomposite, *Geophys. Res. Lett.*, **32**, L18713.
47. Scafetta, N., and West, B.J. (2006) Phenomenological solar signatures in 400 years of reconstructed Northern Hemisp˙ere temperature records, *Geophys. Res. Lett.*, **33**, L17718.
48. Schaefer, J.M., et al., (2006) *Science*, **312,** 1510-1513.
49. Sherwood, S.C., Lazante, J.R., and Meyer, C. L., (2005) Radiosonde daytime biases and late-20th century warming, *Science*, **309**, 1556-59.
50. Thompson, L.G., Mosley-Thompson, E. Brecher, H., Davis, M.E., Leon, B., Les, D., Mashiotta, T.A., Lin, P.-N., and Mountain, K..(2006) Evidence of abrupt tropical climate change: past and present. *Proceedings of the National Academy of Sciences*, 1**03(28),** 10536-10543. http://www-bprc.mps.ohio-state.edu/Icecore/Abstracts/Publications.html
51 Trenberth, K.E., Fasulo, J., and Smith, L. (2005) Trends and variability in column-integrated atmospheric water vapor, *Clim. Dyn*., **24**, 741-758.
52. Velicogna, I. and Wahr, J. (2006) Measurements of Time-Variable Gravity show mass loss in Antarctica, Science Express, www.scienceexpress.org, 10.1126/science.1123785.
53. Vinnikov, K.Y., and Grody, N.C. (2003), Global Warming trend of mean tropospheric temperature observed by satellites, *Science*, **302**, 269-272.
54. von Storch et al., (2004) Reconstructing past climate from noisy data, *Science*, **306,** 697.
55. von Storch et al., (2006) Response to comment on "Reconstructing past climate from noisy data, *Science*, **512**, 529.
56. Wild et al.(2005) From Dimming to Brightening: Decadal Changes in Solar Radiation at Earth's Surface, *Science* , **308**, 847 - 850
57. Willson, R.C., and Mordvinov , A.V. (2003) Secular total solar irradiance trend during solar cycles 21-23, *Geophys. Res. Lett.*, **30**, 1029-1032.

**This article should be cited as follows:**

Keller (2007) Global Warming 2007. *An Update to Global Warming: The Balance of Evidence and Its Policy Implications. TheScientificWorldJOURNAL* **7**, 381–399. DOI 10.1100/tsw.2007.91.
